# Long-term outcomes after repair for anomalous right coronary artery from the pulmonary artery

**DOI:** 10.1017/S1047951122000373

**Published:** 2022-02-18

**Authors:** Andrew Tran, Lazaros Kochilas, Amanda S. Thomas, Varun Aggarwal

**Affiliations:** 1 Emory University School of Medicine, Atlanta, GA, USA; 2 Department of Pediatrics, Emory University School of Medicine, Atlanta, GA, USA; 3 Children’s Healthcare of Atlanta, Atlanta, GA, USA; 4 Division of Pediatric Cardiology, Department of Pediatrics, University of Minnesota Masonic Children’s Hospital, Minneapolis, MN, USA

**Keywords:** Congenital, cardiology, paediatrics, anomalous right coronary artery from the pulmonary artery

## Abstract

Anomalous right coronary artery from pulmonary artery (ARCAPA) is a rare congenital heart disease that can lead to abnormal coronary perfusion and a need for surgical repair. Here, we report the outcomes of patients who underwent ARCAPA surgery within the Pediatric Cardiac Care Consortium (PCCC), a North American registry of interventions for paediatric heart diseases. We queried the PCCC for patients undergoing surgical repair for ARCAPA at <18 years of age between 1982 and 2003. Outcomes were obtained from the PCCC and after linkage with the National Death Index (NDI) and the Organ Procurement and Transplantation Network (OPTN) through 2019. Twenty-four patients (males: 15) were identified having surgery for ARCAPA at a median age of 5.8 (IQR 2.7–10.3) years. Of them, 23 cases were considered “simple” (without major intracardiac disease) and one “complex” (co-existing with tetralogy of Fallot). Five patients presented with symptoms [chest pain (1), dyspnoea on exertion (2) or history of syncope (2)]; while the remaining 19 patients were referred for evaluation of either murmur or co-existing CHD. There was no in-hospital mortality after the surgical repair. Fourteen patients had sufficient identifiers for NDI/OPTN linkage; among them, only one death occurred from unrelated non-cardiac causes within a median period of 19.4 years of follow-up (IQR: 18–24.6). Outcomes were excellent after reimplantation up to 25 years later and further longitudinal monitoring is important to understand the interaction of pre-existing coronary pathology with the effects of ageing.

Anomalous right coronary artery from pulmonary artery (ARCAPA) has not been reported in many epidemiological studies on coronary artery abnormalities.^1–3^ Due to limitations in diagnostic modalities and a lack of general population screening, determining the exact incidence of ARCAPA in the general population is difficult. Yamanaka and Hobbs found two occurrences of ARCAPA out of 126,595 angiography patients and concluded that ARCAPA accounts for 0.002% of all coronary artery abnormalities.^4^ The lesion is characterised by abnormal coronary perfusion due to a “steal phenomenon” from the left coronary artery and via collateral connections to the right coronary artery and into the low-pressure pulmonary artery. Patients with ARCAPA can have varied presentation from asymptomatic to angina, dyspnoea, and palpitations.^5^ ARCAPA has been reported in both children and adults with “bimodal age distribution” of early childhood and later adulthood time of diagnosis.^5^ Surgical reimplantation of the anomalous right coronary artery to the ascending aorta is the standard of care in the management of these patients. Historically, some patients received right coronary artery ligation, but this is sub-optimal and avoided whenever possible.^6,7^ Due to the rarity of the condition, outcomes after surgical ARCAPA repair are limited to isolated case reports or case series with limited follow-up.^5,8,9^ Here, we report the in-hospital and long-term outcomes of patients after surgical repair for ARCAPA who are enrolled in the Pediatric Cardiac Care Consortium (PCCC), a large US-based registry of interventions for CHDs.^10^

## Methods

We queried the PCCC registry for patients operated for ARCAPA at <18 years of age. The PCCC collected data from 47 centres in North America between 1982 and 2011, when it stopped enrolling new patients. This study was approved by the Institutional Review Board of Emory University and by the National Death Index (NDI) and the Organ Procurement and Transplantation Network (OPTN). The registry is supported by the National Heart, Lung, and Blood Institute R01 HL122392 and the Department of Defense (PR180683) grants.

Patient demographic variables, diagnosis, procedure/surgical details, and in-hospital outcomes were abstracted from the PCCC records. Post-discharge outcomes after surgery were obtained from the follow-up notes available in the registry. Survival or transplant data were available for patients that had adequate identifiers for linkage with the US NDI and the OPTN through 2019. Patients with non-US resident status or who were enrolled after the stricter Health Insurance Portability and Accountability Act rules implementation on 15 April 2003, were excluded from the long-term study cohort (NDI-ineligible). The remaining patients were classified as NDI-eligible. Among the NDI-eligible patients, only those with an available identifier were submitted to the NDI (NDI-submitted).

Survival time was calculated from hospital discharge after the patient’s surgical repair for ARCAPA to the date of (1) death, (2) heart transplant, or (3) 31 December 2019 (last date of follow-up provided from the NDI and the OPTN), whichever came first. Underlying and contributing causes of death were obtained from NDI-Plus (a supplement to the NDI dataset containing information on causes of death) and were categorised according to previous reports.^10^

Descriptive statistics are presented as frequency and percentages for categorical variables, while continuous data with non-normal distribution are presented as medians with interquartile range (IQR). Analyses were performed using SAS version 9.4 (Cary, NC).

## Results

**Figure 1.**
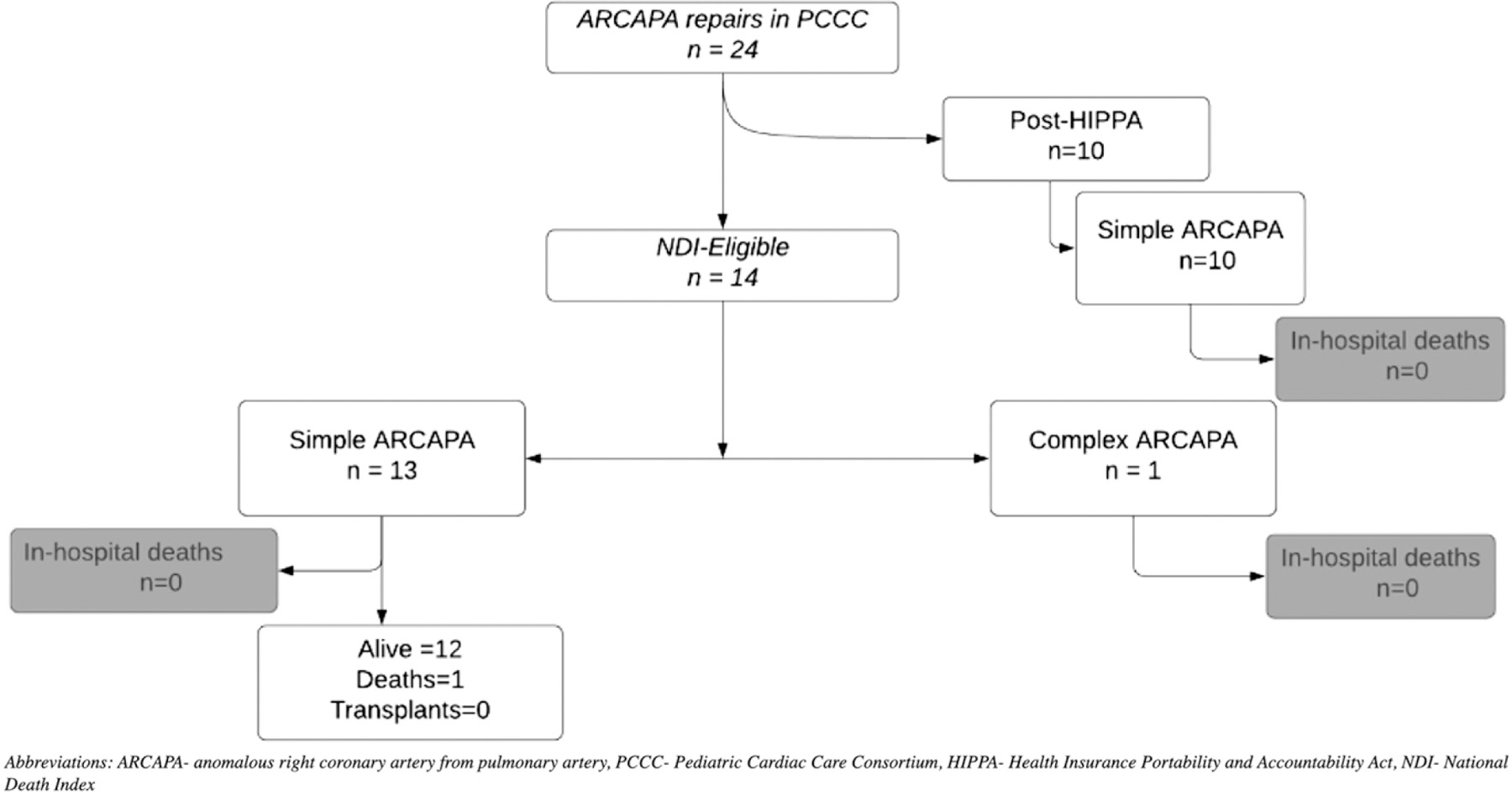
Flowchart of the PCCC cohort with ARCAPA repair and outcomes. ARCAPA = anomalous right coronary artery from pulmonary artery; HIPPA = Health Insurance Portability and Accountability Act; NDI = National Death Index; PCCC = Pediatric Cardiac Care Consortium.

A total of 42 patients in the PCCC registry were diagnosed with ARCAPA accounting for 0.03% among 94,960 patients operated in the PCCC between 1982 and 2011 with 132,048 cardiac operations.^11^ Of them, 13 had a previous surgery for ARCAPA outside the PCCC while five had ARCAPA as part of a complex CHD and did not undergo ARCAPA reimplantation (hypoplastic left heart syndrome n = 3, tricuspid atresia n = 1, D-transposition of the great arteries n = 1). After excluding these patients, 24 underwent reimplantation of the right coronary artery to the aorta and were included in the study cohort. Among them, 23 were classified as simple and included (i) patients with ARCAPA as the only intracardiac lesion (n = 16), (ii) the patients had coexistent simple CHD (ventricular or atrial septal defects, n = 5 and/or patent ductus arteriosus, n = 1; coarctation of aorta, n = 1) (Fig 1). The remaining one patient had associated tetralogy of Fallot (ToF) and was classified as “*complex ARCAPA*” (Fig 1).

Table 1 depicts the characteristics of the PCCC study cohort with ARCAPA. Patients with simple ARCAPA were diagnosed at a median age of 5.6 years (IQR: 2.1–10). 10/23 patients in the simple ARCAPA group were asymptomatic at the time of presentation, and murmur was listed as the indication for the evaluation, while 5/23 of the patients presented with coronary steal-related symptoms such as chest pain (n = 1), dyspnoea on exertion (n = 2), or history of syncopal episodes (n = 2). No information was available regarding the circumstances of diagnosis for three of the patients. The remaining (5/23) were diagnosed as part of the workup for co-existing cardiac lesions, and one patient was diagnosed after surgical repair of coarctation of the aorta. The patient in the complex ARCAPA group presented with a murmur and diagnosed to have ToF with ARCAPA during diagnostic angiography. Most of the patients were diagnosed by angiography (n = 21), while one was diagnosed by echocardiography, and the method of diagnosis was not available in the registry data on one patient. On angiography of the simple ARCAPA cases (21/23), the impressions included reports of “dilated and/or tortuous right coronary artery” that drained into the “main pulmonary artery” and had retrograde filling from collaterals from the left coronary arterial system. On echocardiography (1/23), the ARCAPA originated from the pulmonary artery trunk at the level of the pulmonary valve sinuses. 1 patient did not have an angiography report, but the surgical note mentioned the angiographic diagnosis of right coronary artery coming from the main pulmonary artery.

The patient with the ToF was diagnosed at the age of 3 months with ToF. In addition to the coronary reimplantation, five patients with ASD, VSD, and PDA underwent concomitant closure of the defects and one of them additional pulmonary arterioplasty of main pulmonary artery at the site excision of the ARCAPA. One additional patient had already spontaneous closure of the small muscular VSD, so no VSD intervention was required at the time of the coronary reimplantation. The patient with the ToF underwent transatrial repair and placement of epicardial pacemaker at the age of 9 months for persistent heart block. The ARCAPA was not reimplanted until 10 years later at which time the pacemaker was removed, because the atrioventricular conduction was restored and there were no symptoms of ischaemia.

Among the 14 patients with available identifiers (NDI-eligible and NDI-submitted), there was no transplant and only one death over a median follow-up duration of 19.4 years (IQR: 18–24.6). The death occurred 10 years post-reimplantation of a simple ARCAPA case. The details of the cause of death were not available in the registry database. Follow-up in the PCCC with angiography or echocardiography was available in six patients (all with simple ARCAPA). One patient who had a simple ARCAPA received an elective pericardial window for persistent pericardial effusion 2 months after reimplantation surgery. Four patients with simple ARCAPA had echocardiographic data available at their last follow-up. Two of them had RCA dilation, but all were reported to have normal biventricular function. Six patients, including the four patients with echocardiographic data, had a follow-up angiography with median follow-up time 1.56 (IQR 0.05–33.75) months; four of them had RCA (n = 3) or LCA (n = 1) dilation, and none had coronary artery stenosis or ventricular dysfunction. Two of the patients with just echocardiograph had the post-surgical study done before being discharged after the cardiac surgery.

## Discussion

ARCAPA is rarely reported in the paediatric population, and there are only few reports on its clinical presentation, treatment, and course after its surgical repair. So far, only case reports or small series have been reported for children operated for ARCAPA.^8,9,12–17^ Table 2 provides a summary of the prior reports in the literature on children with ARCAPA reimplantation and follow-up. To our knowledge, this report presents the largest cohort of children treated for ARCAPA with and without co-existing CHD and for the longest follow-up time.

Our patient cohort suggests that many patients with ARCAPA can be asymptomatic or have non-specific symptoms (chest pain, syncope, dyspnoea on exertion), and therefore a high index of suspicion is recommended. Patients with co-existing CHD were diagnosed during the diagnostic workup of their co-existing CHD. This is similar to the findings in the review performed by Guenther et al^18^ who reported that 38% of patients were asymptomatic most commonly identified during evaluation of a murmur. The median age of presentation in our cohort was 5.6 years. Guenther et al^18^ reported a bimodal distribution of the age at presentation with one peak centred near birth and another peak centred around 40–60 years of age. We may have missed this bimodal peak in our cohort due to the relatively small numbers. This bimodal peak can be explained due to the underlying pathophysiology in patients with ARCAPA. High pulmonary pressure causes antegrade flow of deoxygenated blood through the right coronary artery in patients with ARCAPA shortly after birth. Blood flows retrograde into the pulmonary artery as child grows older and the pulmonary pressure drops,^19^ resulting in “coronary steal.” The patient may present with symptoms associated with ischaemia in the RCA territory during the newborn period if collateralisation between the left and right coronary arteries has not matured adequately, as demonstrated by the first peak in the distribution of symptomatic patients. The patient’s left coronary artery distributes oxygenated blood to the entire heart if collateralisation between the left and right coronary artery is present, and pulmonary pressure remains low, and then, the patient may continue into adulthood without symptoms. Nevertheless, the myocardial perfusion abnormalities seem to be of lesser impact than in the case of ALCAPA at the time of diagnosis as none of the patients in our cohort had evidence of myocardial ischaemia or atrioventricular valve dysfunction. Similar to previous reports,^18^ conventional cardiac angiography was the most prevalent method of diagnosis in patients with ARCAPA. Retrograde flow via the RCA, indications of substantial collateralisation between the left and right coronary artery systems, increased flow with left and right coronary artery dilatation, and direct flow between the right coronary artery, and the pulmonary artery are all known angiographic hallmarks of ARCAPA.^20^

We demonstrate that reimplantation of the anomalous RCA has excellent operative outcomes up to 25 years post-repair. Beyond survival, the limited follow-up data in the PCCC revealed coronary artery dilation in four of the six patients with cardiac catheterisation, probably reflecting the exposure to increased coronary flow prior to the surgical reimplantation. Other studies have reported similar findings after reimplantation surgery and described the anomalous RCA as being “thin-walled and vein-like in character,” so its diameter is less likely to return to normal after surgery.^21^ This dilation was not associated with any known ventricular dysfunction nor any other complications in our series, although there is report of a clot formation in this setting.^9^ Kinking of the reimplanted RCA has been described in the literature, but this was not observed among the patients with available follow-up in our cohort._15_ We do not have enough patients with ARCAPA reimplantation in the context of complex CHD to meaningfully report on their long-term outcomes. However, given the benign course of the reimplanted ARCAPA in the simple cases, we expect that their outcomes will likely be defined mostly by the underlying CHD.^22^ One patient with ToF had evidence of complete heart block post-surgical repair of ToF. Albeit coronary perfusion abnormalities in the distribution of the RCA may contribute to the heart block; in our patient, it is likely related to the surgical repair of ToF. This review emphasises the lack of long-term data after ARCAPA repair as the few existing reports are limited to only a few adult patients with short-term follow-up.^8,23,24^ Our study demonstrates how a long-standing clinical registry can provide long-term outcomes for even rare conditions such as ARCAPA reimplantation after linkage with national event registries.

Although the PCCC contains a wealth of information, it is still a limited and retrospective data source that contains data from various sources for an extended period of time. Some notes that were scanned into the PCCC were also handwritten, which leaves room for misinterpretation. Despite being a large registry including patients from many centres across the USA, we had relatively small cohort of patients due to the rarity of the condition. In addition, we analysed a historical cohort with operations performed between 1982 and 2011, and with advancement in surgical expertise and the improvement in the peri-operative care; even better outcomes than seen in the registry may be anticipated in the current era. Regardless of the limitations, this study is important in highlighting the excellent outcomes with the surgical repair of ARCAPA so that corrective surgery should not be behold from children identified with this rare anomaly.

## Conclusion

Patients with ARCAPA have excellent outcomes after reimplantation surgery up to 25 years later. Monitoring these patients longitudinally will be important to understand the interaction of pre-existing coronary pathology and post-surgical conformational changes with the effects of ageing.

## Figures and Tables

**Table 1.** Characteristics of patients with ARCAPA reimplantation in the PCCC.

	Simple*	Complex**
Total (N)	23	1
Gender		
Male	15	0
Female	8	1
Median age at diagnosis, years (IQR)	5.6 (2.1–10)	0.2
Median age at reimplantation, years (IQR)	5.2 (2.2–10.2)	10.3
In-hospital deaths	0	0
NDI-submitted	13	1
Deaths among NDI-submitted	1	0

* Simple cases include atrial septal defects (ASDs), ventricular septal defects, patent ductus arteriosus (PDA), patent foramen ovale (PFOs) only in addition to ARCAPA surgical intervention

** Patient had Tetralogy of Fallot repair at 9 months of age and ARCAPA reimplantation at 10 years of age

**Table 2.** Prior reports on children with ARCAPA reimplantation.

Author	Age at diagnosis (years)	Sex	Type of case	Presenting symptoms	In-hospital outcomes	Follow-up information
Achtel (1975)^25^	10	M	Simple	Murmur and cyanosis	Discharged	1 year: RCA patent
Lerberg (1979)^26^	6	M	Simple	Fatigue	Discharged	4 years: No events
Guenther et al^18^	13	M	Simple	–	Discharged	3 years: No events
Lawrence Moss^16^	8	F	Complex	CHD: ToF	Discharged	1 year: No events
Van Meurs-Van Woezik et al^17^	0.3	F	Simple	Murmur	Discharged	–
	9	F	Simple	Chest pain	Discharged	–
	0.3	F	Complex	CHD: ToF	Discharged	–
	3.5	F	Simple	Murmur	Discharged	6 years: RCA clot
	0.1	M	Complex	CHD: ToF/PA	Death	
	4	F	Complex	CHD: CoA	Discharged	–
	8	M	Simple	Murmur	Discharged	–
Chernogrivov et al^12^	1	F	Simple	Murmur	Discharged	2 years: No events
Parasramka (2011)^27^	21	M	Simple	Chest pain and dyspnoea	–	
Mahdavi (2013)^28^	0.17	M	Simple	Heart failure	Discharged	–
McCracken et al^10^	17	M	Simple	–	Discharged	3 months: RCA patent
Guenther et al^5^	0.67	M	Simple	Seizures	Discharged	3 years: No events
	1.5	M	Simple	Murmur	Discharged	1 year: No events
	4	M	Simple	Abnormal ECG	Discharged	6 months: No events
Thammineni et al^11^	0.08	F	Simple	Murmur	Discharged	15 months: No events
	1.5	M	Simple	Murmur	Discharged	7 months: No events

CHD = congenital heart defect; CoA = coarctation of the aorta; F = female; M = male; PA = pulmonary atresia; RCA = right coronary artery; ToF = Tetralogy of Fallot
